# A review of implementation aspects and sustainability in the prevention of hospital onset bacteremia

**DOI:** 10.1017/ash.2025.1

**Published:** 2025-02-24

**Authors:** Robert Garcia, Edward J. Septimus, Jack LeDonne, Lisa K. Sturm, Nancy Moureau, Michelle DeVries, Barbara DeBaun

**Affiliations:** 1 Enhanced Epidemiology LLC, Valley Stream, NY, USA; 2 Department of Population Medicine, Harvard Medical School, Boston, MA, USA; 3 Chesapeake Vascular Access, Baltimore, MD, USA; 4 Ascension, St. Louis, MO, USA; 5 Griffith University and PICC Excellence, Inc., Hartwell, GA, USA; 6 ICU Medical, San Clemente, CA, USA; 7 B. DeBaun Consultants, San Francisco, CA, USA

## Abstract

The emerging perspectives and implementation aspects presented in this review article outline infection prevention core components supported by recent research relevant to the mitigation of Hospital Onset Bacteremia and Fungemia in a surveillance setting that includes expanded efforts to all vascular access devices.

## Background

A proposed revision by the Centers for Medicare and Medicaid Services (CMS) to mitigate Hospital Onset Bacteremia and Fungemia (HOB) associated with all sources including all vascular access devices (VAD), requires system-wide coordination to effectively expand a more universal approach to surveillance and prevention capabilities.^1^ Aspects to influence future decisions in the modification of infection prevention (IP) programs directed toward this federal regulatory revision include both technical and implementation considerations. Technical aspects in VAD HOB prevention have been addressed in a recent publication.^2^ The implementation and sustainability aspects, outlined here by infection preventionists, infectious disease (ID), and vascular access specialists (VAS), are intended to provide insights and illuminate emerging perspectives for enhancement of HOB preventive practices in the acute care hospital setting. *Implementation Guide* sub-sections provide principal guideline references. Whereas the *Execution Process* sub-sections will outline BSI prevention projects and studies where applicable that provide real-world tactics relevant to the early development in the prevention of HOB.

## Implementation core components

### Leadership

#### Implementation guide

Prevention of HOB achieves wider success with leadership engagement, rather than implementation of clinical “bundle” approaches alone. A study conducted in US hospitals and examined management roles directed at the prevention of central line-associated BSI (CLABSI), indicating three specific HAI prevention practices central to HAI prevention efforts: engagement of executive and frontline manager leadership, information sharing, and manager coaching.^3^

#### Execution process

Key components of a Comprehensive Unit-Based Safety Program (CUSP) initiative to prevent CLABSI in intensive care units (ICUs) in two US hospitals included engagement from leadership and physicians. Hospital leadership provided support to identify an executive champion for each participating ICU. CLABSI rates decreased by 84% over a six-year period. The study provides support that leadership-directed culture change, communication, and teamwork create improvements in patient care and may assist in the transition to broader-scale harm reduction with HOB.^4^

### Staffing

#### Implementation guide

HOB prevention requires IP leaders to add potential hazards to their annual risk assessment. Using this added information, coupled with information gathered from audits and observations,^5^ will help examine staffing structures, skill level of IP staff, and resource allocation. The goal is to revise job descriptions, adjust career ladders, and establish supportive roles^6^ that define new staff functions focused on preventing HOB and primary infection sources. Additional strategies that support and assist in increasing IP program capacity are outlined in “Ten Pillars” that focus on structure, processes, empowerment, and partnerships.^7^

Recent advancements have led to more precise systems for assessing IP staffing needs as demand grows across diverse healthcare settings. The APIC Calculator aids in determining effective IP staffing by factoring in care complexity, facility size, patient case mix, surveillance workload, laboratory and procedure volumes, data and reporting demands, and external program responsibilities.^8^ Additionally, the Infusion Nurses Society Standards of Practice provides key guidelines for structuring and staffing a vascular access team (VAT).^9^

#### Execution process

An online survey-based study used to validate the APIC calculator indicated that 79.2% of respondent’s staffing levels were below expected. In addition, IP staffing levels below expected showed much higher rates of HAIs including CLABSIs.^10^ Data suggests that hospitals with VATs report greater adherence to evidence-based practices and experience lower rates of device-associated complications such as infection and deep vein thrombosis (DVT).^11^ A survey on the structure and function of VATs reveals that hospitals without such teams most commonly cite limited resources, insufficient staff, or inadequate patient volume as reasons for not establishing a VAT.^11^

### Integration of hospital epidemiology and infection prevention

#### Implementation guide

In response to the growing complexity and sophistication of IP, a new paradigm has emerged that advocates the integration of IP programs with Hospital Epidemiology (HE).^12^ The advantages of a unified department include a multidisciplinary approach to IP activities, rapid access to specialized expertise, and coordinated application of IP knowledge across disciplines, enhancing the ability to reduce HAIs like HOB. A survey conducted in United States-based System Healthcare Infection Prevention Programs (SHIPPs) institutions has illuminated the next steps in defining optimal structure and resources needed by physician directors for expanding service across healthcare systems. Healthcare facility types included critical access hospitals, long-term care acute care hospitals, nursing homes, and academic centers. The data provides insight on physician salaries, consult coverage, site visits, roles in addition to IP responsibilities, and IP resources. The biggest challenges in expanding integration included gaps in clear governing structure, communication across the system, consistent staffing with empowered IP experts, and data management support. The authors recommend the drafting of a white paper addressing system healthcare IP.^13^ Optimization of ID integration across distinct types and sizes of healthcare facilities in network systems assists in the effectiveness of HOB prevention programs.

#### Execution process

Studies are needed to evaluate the impact of integrating ID/HE into IP programs.

### Artificial intelligence and infection surveillance

#### Implementation guide

IP programs are in the initial stages of transitioning infection surveillance from manual review methods to fully electronic reporting systems that utilize data from electronic health records (EHRs). The introduction of artificial intelligence (AI) and machine learning systems will enhance laboratory-based diagnosis and antimicrobial resistance detection, while also aiding in the prediction, prevention, and risk stratification of HOB events.^14^

#### Execution process

AI as it applies to VAD HOB will require refinements that consider variabilities in EHR documentation (eg, documentation of patient signs and symptoms recorded in non-standardized ways).^15^ A recent proof-of-concept study examined the effect of using AI technology to identify cases of HAIs in complex clinical scenarios. With clear prompts, AI tools accurately identified an HAI in six fictional patient scenarios of varying complexity. However, researchers noted that missing or ambiguous information in the descriptions could hinder the AI’s ability to produce accurate results.^16^ While AI offers significant potential benefits for IP—such as enhancing workflows for infection preventionists and helping hospitals prioritize IP efforts—challenges remain, including reducing false positive HOB events and improving applicability in diverse clinical settings.

### Diagnostic stewardship and blood culture management

Proper ordering and collection of blood cultures (BCs) significantly enhance HOB surveillance by ensuring the recovery of true pathogens (avoiding false negatives), improving the accuracy of VAD-associated BSI event monitoring, and preventing blood culture contamination (BCC).^17^ BCC sets off a cascade of serious consequences with global implications for hospitals. These include inappropriate antimicrobial treatment, which can lead to adverse drug reactions, the rise of antibiotic-resistant organisms (AROs), microbiome disruption causing *Clostridioides difficile* infections, unnecessary testing, increased laboratory and pharmaceutical costs^18^ and potential financial penalties or lost reimbursements due to misclassified reportable HAIs.^19^

#### Implementation guide

To achieve a full spectrum of benefits in VAD HOB prevention, hospitals must consider the scientific evidence that supports the optimization of specimen ordering protocols, standardized procedures, and education of personnel in the collection of laboratory specimens, as well as in the reporting and accurate interpretation of laboratory findings. This principle, known as Diagnostic Stewardship (DS),^20^ applies to the pre-analytic phase of procedures including collection of blood for microbiologic culture, therefore giving credence to the need to establish an evidence-based *blood culture management* program as a fundamental step in the accurate identification of BSIs.^17^

#### Execution process

1. *Establish Evidence-Based Decision Aids.* Most BCs are negative (∼90%) and 24-40% of positive BCs detect only contaminants. A key first step in optimizing the accuracy of BC results is establishing a decision-making process to guide clinicians on *when* to order BCs. Compelling evidence exists to support this contention. In the DISTRIBUTE (DIagnostic STewaRdship Improves Blood cUlTurEs) quality improvement study, an algorithm was developed based on the probability of bacteremia (high, moderate, or low). BCs were not recommended for scenarios with a low probability of bacteremia.^21^ Participants received feedback on BC rates and the appropriateness of their decisions. This study found that implementing BC ordering practices in a medical ICU and medicine wards at a large academic center reduced BC utilization by 18% and 30%, respectively.
2. *Standardize evidence-based methods for proper collection of BCs.* Hospitals should review and standardize *how* BCs are collected throughout an organization. Strategies include limiting collection from intravascular catheters, proper skin antisepsis, drawing the correct volume of blood, and drawing the appropriate number of BC sets.^18,22^ Implementing a multidisciplinary intervention model based on Clinical and Laboratory Standards Institute (CLSI) guidelines at a large tertiary hospital resulted in significant improvements in reduced BCC from 1.4% to 0.9%.^23^
3. *Consider blood diversion.* Diversion of the first portion of blood theoretically removes contaminating organisms that survive after local skin disinfection from the remaining aliquot of blood. Nine studies cited in a systematic review and meta-analysis indicated that unifying proper drawing techniques and diversion devices compared with a standard procedure of collection resulted in reduced BCC rates ranging from 0.0% to 2.6%.^24^ It is important to determine the cost-benefit of such modifications in scenarios where interventions involve new devices.

### Human factors engineering

#### Implementation guide

Human factors engineering (HFE) “…is the scientific discipline concerned with understanding the interactions among humans and other elements of a system to improve system performance and well-being”.^25^ HFE considers the *complexity* of modern healthcare (eg, the number of VAD inserters, types of catheters, catheter access) and the *ambiguity* or uncertainty of the system (eg, the skill levels of VAD inserters, compliance with elements of a prevention bundle).

#### Execution process

Although HFE has seen limited application to IP, there are examples that demonstrate benefit after application of HFE models to IP challenges. Researchers examined the influence of an HFE-based intervention that focused on behavior-shaping factors with a goal to increase adherence to best practices and reduce CLABSI events. Maintenance kits were designed using a framework that incorporated seven principles aimed at increasing adherence. Standardization of tools over a 29-month period led to a significant reduction in the number of CLABSI.^26^

### Approaches for prevention

#### Implementation guide

How the infection preventionist *translates* recommended interventions into *actual* practice is fundamental in achieving successful outcomes. The National Quality Forum (NQF) has developed a guidebook with action areas in four phases that address HOB prevention, identification, and treatment. The publication “presents considerations and best practices so that organizations have options that can be tailored to the specific needs of their patients and families, clinical care teams, and acute care settings.”^27^ In an attempt to advance the quality of care and improve patient safety the Association for Vascular Access (AVA) along with various stakeholders has published a consolidation of standards of practice into a comprehensive document related to peripheral intravenous catheters (PIVC).^28^

#### Execution process

Success in application of practices and interventions using IP implementation concepts and frameworks varies widely depending on organization factors such as operational support, data integration, resource allocation, willingness to change, and safety culture.^29^ Strategies designed to reduce primary BSI associated with non-central line VADs are highlighted in a study conducted in a large U.S. based hospital. Creation of a multi-modal initiative and maintenance bundle addressing PIVCs, which included proper assessment of insertion sites, removal of catheters when there is an indication of phlebitis, dressing assessment, needleless connector interventions, and minimizing IV tubing disconnections, resulted in BSI reductions from 0.57/1000 patient days to 0.11/1000 patient days over a 7-month period.^30^ Prevention of *primary* sources to prevent *secondary* BSI, eg, both non-ventilator and ventilator associated pneumonia, skin/soft tissue, wound, urinary tract, and surgical site infections will require a need to revise frameworks for review of HOB cases and expand the notions of prevention.^31^

### Bundle compliance

#### Implementation guide

Drafting, dissemination, and education of IP protocols alone is often insufficient in achieving sustained improvements. Long-term success requires measuring the level of *compliance with individual process components* that comprise intervention bundles, followed by identifying barriers related to specific elements that are deemed below acceptable standards.

Measuring compliance requires quality improvement and IP departments to extract relevant information from the institutional electronic medical record (EMR). Periodic EMR reviews identify “non-compliant” evidence-based bundle elements essential to infection risk reduction, eg, daily chlorhexidine gluconate (CHG) skin decolonization in patients with central VADs.^32^ Validation of information within EMRs helps in avoidance of misguided interventions by identifying limitations of documentation.

**Figure 1.**
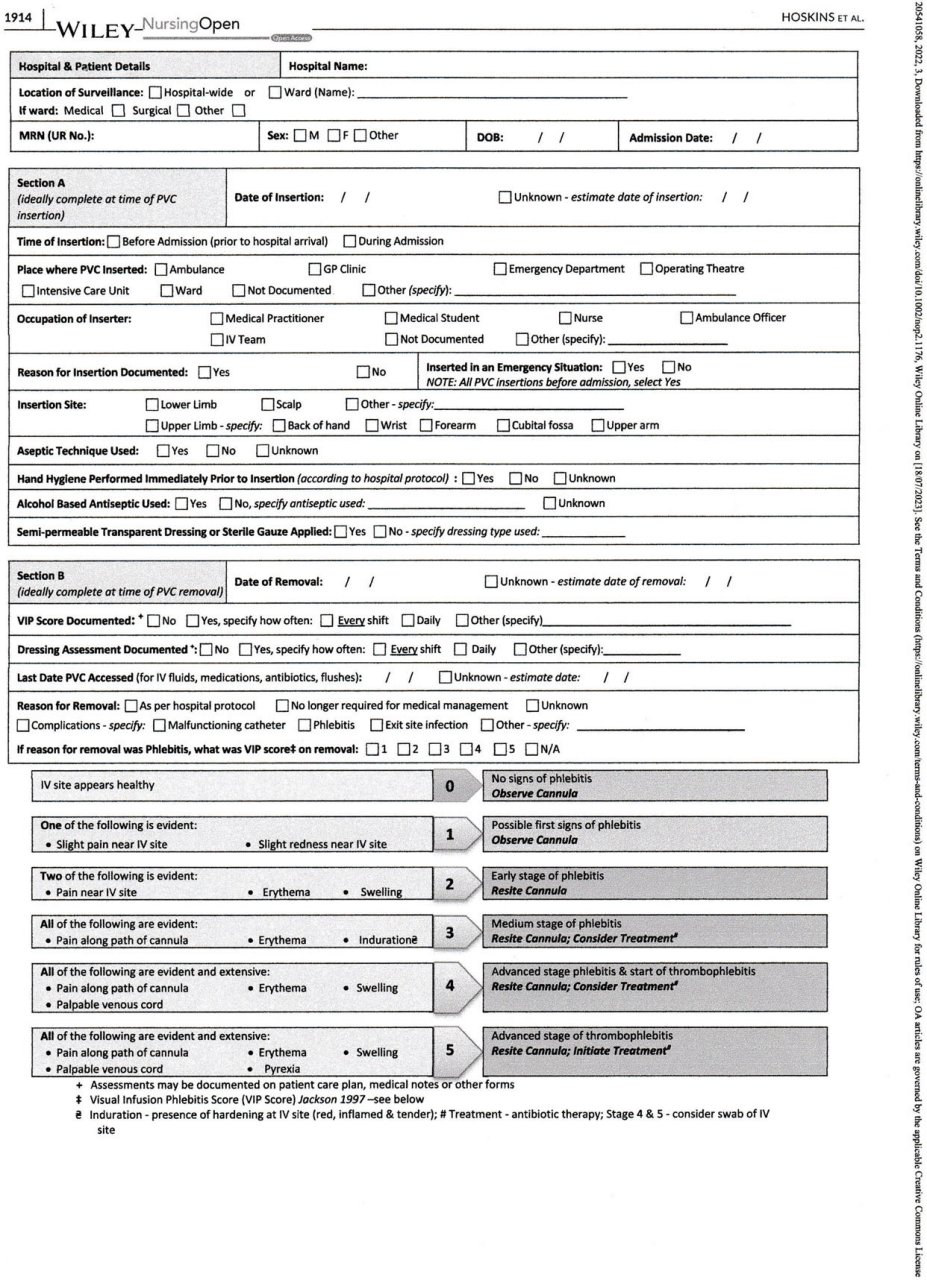
Point prevalence audit tool for PIVCs. (used by permission, Hoskins 2022).

Standardized audit tools are useful in evaluating practices related to VAD insertion, maintenance, and removal. Data compiled in an audit tool should address date and time of insertion, inserter, reason for insertion, whether aseptic technique was performed, date of removal, phlebitis scores, daily dressing assessments, and reason for removal. (Figure 1)^33^

#### Execution process

The need to achieve compliance with individual process components is well supported in a study conducted in 984 ICUs whereby reductions in CLABSI events were not related to bundle implementation but rather when a ≥95% compliance level with bundle components was reached.^34^ Further studies are needed in the application of bundles and compliance to PIVCs and other VADs.

### Data comprehension and practice change

#### Implementation guide

Despite effective audit and feedback of quality data, inconsistencies exist in responses related to clinician behavior. As data comprehension may be a potential factor in the prevention of HOB, a scale has been developed to assess comprehension of BSI quality metric data. The tool has HOB policy relevance by aiding efforts to make quality metrics more effective in influencing medical decision-making and *promoting necessary practice changes*.^35^

#### Execution process

An evaluative study used an 11-item comprehension instrument that contained questions related to metric assessment (eg, which is better: a higher or lower Standardized Infection Ratio?).^35^ Using the information tallied from this survey answered by ninety-seven clinician respondents, the researchers identified specific factors that when modified into a comprehension scale may prove to be most relevant in driving practice change. The understanding of metric data by clinicians and other healthcare leaders will become increasingly important in promoting IP in an expanded surveillance environment.

### Policy

#### Implementation guide

Assessment on the potential areas of focus for HOB event preventability indicated the most common sources to be endovascular (47%; nearly all intravascular catheter-related infection), gastrointestinal (18%), urinary (13%), respiratory tract (13%), and surgical site infection (9%).^36^ Healthcare policy on such prevention endeavors as HOB entails a multi-disciplinary approach including evidence-based practices published in expert guidelines and peer-reviewed literature. Reference can be made to the table “S*ynthesis of Selected Technical Aspects of Vascular Access Devices*” in a recently published article addressing the prevention of HOB associated with all VADs.^2^

#### Execution process

Aseptic non-touch technique (ANTT) is a standardized practice that has become widely utilized and emphasizes fundamental infection prevention rules including hand hygiene and protection of key parts and sites from contact. A study was conducted in a low- and middle-income country to evaluate the adaptation of ANTT implementation for reducing CLABSIs in a pediatric population.^37^ ANTT adherence of 95% was achieved after 6 plan-do-study-act (PDSA) cycles. The CLABSI rate decreased from 5.7 to 1.26/1000 CD.

### Education of VA specialists and infection preventionists

#### Implementation guide

The VAS serve their organizations as consultants and technical inserters with the primary goal of ensuring safe, reliable vascular access to promote long-term vessel health and preservation. Optimal vascular insertion and maintenance requires clinicians to obtain appropriate education. Key points regarding VAS training, education, and competency have been detailed.^38^ No specific IP training resource addressing VADs is currently available.

#### Execution process

VAS, as well as infection preventionists they collaborate with, demonstrate their foundational knowledge of the field through achievement of certification. Certification through the Vascular Access Certification Corporation, VA-BC board certification validates achievement of knowledge.^39^ The credential is not limited to only those persons who physically insert the devices. Obtaining vascular access certification by IPs will assist in the analysis of HOB events related to VADs. Strategies to address strengthening IP practices include regularly auditing VAD procedures, dressings for adherence, and providing ongoing education on aseptic management and infection prevention practices, amongst other quality interventions.^40^

## Conclusion

**Figure 2.**
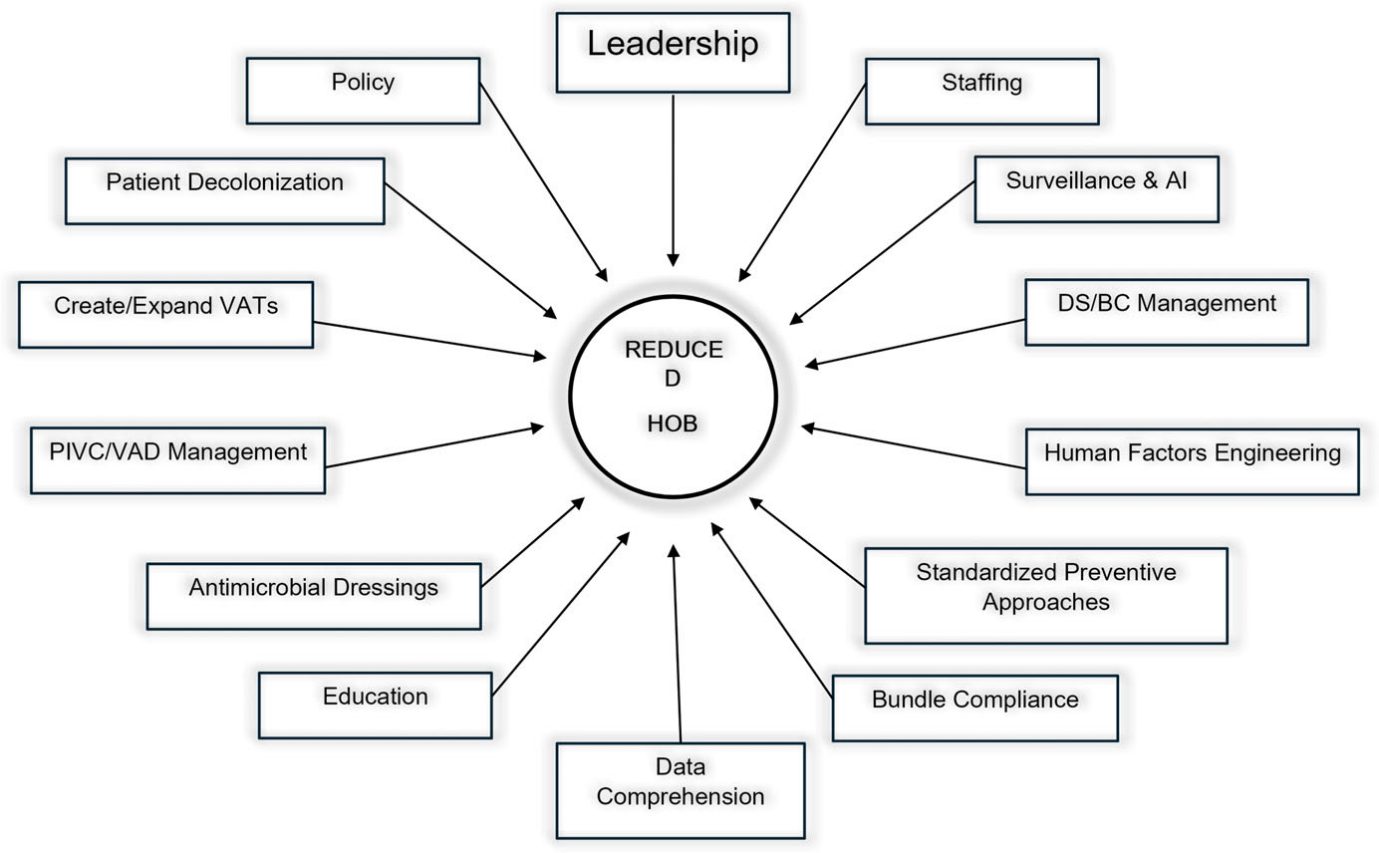
Core components of an HOB prevention program.

The evolution of BSI prevention in hospitals from a narrow CLABSI surveillance focus to a much wider universal all-cause approach will require the need for a broader promotion of universal prevention strategies across all disciplines. This article outlines core components (Figure 2) encompassing implementation, examples of execution, and sustainability strategies that address mitigation of BSIs including those associated with all VADs.
